# Identification of Conjugated Dienes of Fatty Acids in *Vischeria* sp. IPPAS C-70 under Oxidative Stress

**DOI:** 10.3390/ijms25063239

**Published:** 2024-03-13

**Authors:** Roman A. Sidorov, Alexander Y. Starikov, Maria A. Sinetova, Elizaveta V. Guilmisarian, Dmitry A. Los

**Affiliations:** K.A. Timiryazev Institute of Plant Physiology, Russian Academy of Sciences, Botanicheskaya Street 35, 127276 Moscow, Russia; roman.sidorov@mail.ru (R.A.S.); starikovay1393@gmail.com (A.Y.S.); maria.sinetova@mail.ru (M.A.S.); liza.gyulmisaryan@yandex.ru (E.V.G.)

**Keywords:** allene, conjugated dienes, fatty acids, microalgae, oxidative stress

## Abstract

The microalgae *Vischeria* sp. IPPAS C-70 produces eicosapentaenoic acid. Several stresses cause the formation of fatty acid peaks that resemble hexadecadienoic acids. We used the integrated technique including TLC, HPLC, and GC–MS to search and determine these fatty acids. Double bond positioning in these fatty acids indicated that they were conjugated dienes and allenes. We identified and described natural nine isomers of C16 polyunsaturated fatty acids, including common methylene-interrupted dienes (Δ6,9-16:2, Δ7,10-16:2, Δ9,12-16:2), and unusual conjugated dienes (Δ6,8-, Δ7,9-, Δ8,10-, Δ9,11-, and Δ10,12-16:2), as well as allenic diene (Δ9,10-16:2). We hypothesize that the formation of conjugated dienes and allenes among fatty acids is the result of oxidative stress caused by H_2_O_2_. Hydrogen peroxide also caused an increase in saturated at the expense of unsaturated fatty acids, suggesting inhibition either fatty acid desaturases activities or the corresponding gene expression.

## 1. Introduction

Previously, we tested a number of strains from the Collection of Microalgae and Cyanobacteria IPPAS (K.A. Timiryazev Institute of Plant Physiology RAS, Moscow, Russia) to discover potential producers of nutraceutical compounds including fatty acids [1]. That research identified numerous algal strains that generate long chain polyunsaturated fatty acids (LC-PUFAs). *Vischeria* sp. IPPAS C-70, an Eustigmatophyceae strain, accumulates oil droplets containing eicosapentaenoic acid (EPA) [1]. In addition, numerous unique isomers of hexadecadienoic acid were discovered during the adjustment of growth conditions for *Vischeria* sp. The emergence of those molecules was linked to several stresses (growth medium alterations, excessive salt content, culture aging, or oxidative stress). We assumed that these diene isomers could represent conjugated or cumulated dienes (allenes).

The presence of conjugated double bonds in FA acyls alters the physical properties of lipids containing them, which may help algae and higher plants adapt to demanding environments (for example, by dissipating intense light flux in high-altitude terrains [2]). Conjugated FAs appear to be a viable building material for liposome synthesis, which, in addition to their fundamental applications [3], are being intensively studied as transporters for targeted drug delivery [4], including cancer treatment [5]. Conjugated linoleic acid (CLA) has various advantages over common unsaturated FAs [6]. Another conjugated isomer, catalpic acid ((9E,11E,13Z)-octadeca-9,11,13-trienoic acid), controls the accumulation of fat [7]. Punicic acid ((9Z,11E,13Z)-octadeca-9,11,13-trienoic acid) has high antioxidative and anti-inflammatory effects and reduces neurological disorders’ symptoms [8].

Allenes are organic compounds in which one carbon atom has double bonds with each of its two adjacent carbon atoms. Allenic FAs are relatively uncommon molecules; approximately 200 natural compounds having allenic bonds have been discovered. Glutinic acid (HOОС-СН=С=СН-СООН) is a substrate for the synthesis of natural allenes. Laballenic (5R,6-octadecadienoic acid), lamenallenic (5R,6,16E-octadecatrienoic acid), and phlomic (7,8-eicosadienoic acid) acids are abundant in the seeds of various Lamiaceae species [9,10]. Compounds containing allene bonds demonstrated antimicrobial and antiviral activity [11], implying that FAs with allene bonds could have comparable effects. Although the allenic FAs have been reported mostly in higher plants capable of various types of FA modifications, e.g., hydroxylation, epoxygenation, triple-bond formation, or conjugated double bond biosynthesis [12], a limited number of articles mention these molecules found in microalgae as minor lipid components [13]. In addition, the allenic FA puna’auic acid (12,13-dihydroxyoctadeca-9,10-dienoic acid) had been identified in the marine cyanobacterium, *Pseudoanabaena* sp. [14].

Microalgae offer a prospective source of novel FAs and lipids for nutraceutical and medical applications [13,15,16]. Thus, searching for the most effective producers or modifying cultivation conditions to increase target metabolite production is not only practical, but also critical in discovering strategies to control and, therefore, better understand lipid metabolism. For these reasons, the correct FA identification is critical. In ordinary practice, the most frequent approach is gas–liquid chromatography using 60–100 m capillary columns with a highly polar stationary phase, followed by MS detection of fatty acid methyl esters (FAMEs). The identification is based on the coincidence of their mass spectra with those of an NIST-type library. However, this method may not always be sufficient for accurately identifying all FA isomers that may be encountered in practice [17]. For example, the NIST database has mass spectra of isomers of octadecadienoic acids that differ only in the position of the double bond. This is a fairly small group of isomers, consisting primarily of methylene-separated ones that differ from one another in the location of bonds by two or more carbon atoms (6,9-, 9,12-, 12,15-) and some conjugated dienes (CLA).

A similar issue applies to hexadecadienoic FAs. As a result, simple automatic searches for the best match between the researched spectrum and the library spectra can lead to incorrect outcomes.

In addition to MS data, other parameters such as relative retention time, equivalent chain length methodology (ECL) [18], UV spectroscopy, and mass spectrometry of nitrogen-containing derivatives of various types should be considered to accurately determine the carbon number, position, and configurations of double bonds. Therefore, an integrated approach to FA identification is desirable, as relying on a single method can lead to erroneous conclusions. Here, we used the integrated technique and identified, for the first time, nine isomers of hexadecadienoic acid with three distinct types of double bond configurations in eustigmatophycean microalga *Vischeria* sp.

## 2. Results

### 2.1. Fatty Acids of Total Lipids from Cells Cultivated in Tamiya Medium and BBM-3N Medium Supplemented with Hydrogen Peroxide

Table 1 shows the GC–MS chromatography results for sample cells cultured in either 3N Bold’s Basal Medium BBM-3N (control), Tamiya medium for 10 days, or BBM-3N supplemented with 5 mM H_2_O_2_ for 24 h. Section 4.1 provides a description of media composition. The total lipids of control cells of *Vischeria* sp. IPPAS C-70 contain fifteen distinct FAs. In all experimental versions, the major FAs were myristic, palmitoleic, oleic, linoleic, arachidonic, and eicosapentaenoic. Minor acids discovered in all forms included lauric, palmitovaccenic, palmitolinoleic, *cis*-vaccenic, linolenic, arachidic, and eicosatrienoic acids.

We first discovered various unusual isomers of hexadecadienoic and octadecadienoic acids in cells of *Vischeria* sp. strain IPPAS C-70 cultured in Tamiya medium for 10 days, whereas such chemicals were lacking in cells grown in “standard” BBM-3N media (Table 1). Tamiya medium is rich in nitrogen, phosphorus, magnesium, and sulfur. It was successfully used to increase the biomass production of *Chlorella* strains; however, it was shown to be inefficient for *Vischeria* sp. strain IPPAS C-70 [1]. Tamiya medium featured a longer lag phase, a slower growth rate, and a higher concentration of carotenoids among pigments than the “standard” BBM-3N medium. Growth inhibition and increased relative carotenoid content are classic indications of oxidative stress, which normally accompany almost all abiotic stresses in photosynthetic organisms [19,20]. Thus, we sought to see if adding H_2_O_2_ to “traditional” BBM-3N media caused the emergence of unique isomers of dienoic fatty acids.

After incubation in BBM-3N medium with 5 mM H_2_O_2_ for 24 h, a significant number (28.5% of total FA) of unusual isomers of hexadecadienoic acid was detected, which was even higher than that in Tamiya medium (8.1%); the number of isomers of octadecadienoic acids was also higher (Table 1). In addition, hydrogen peroxide significantly increased saturated FAs at the expense of unsaturated species. The amount of palmitoleic acid dropped from 38 to 17%, whereas palmitic acid increased from 23 to 31%. The amounts of oleic, linoleic, arachidonic, and eicosapentaenoic acids also dropped under H_2_O_2_ treatment, suggesting that hydrogen peroxide may affect FA desaturation, but not elongation.

**Figure 1:**
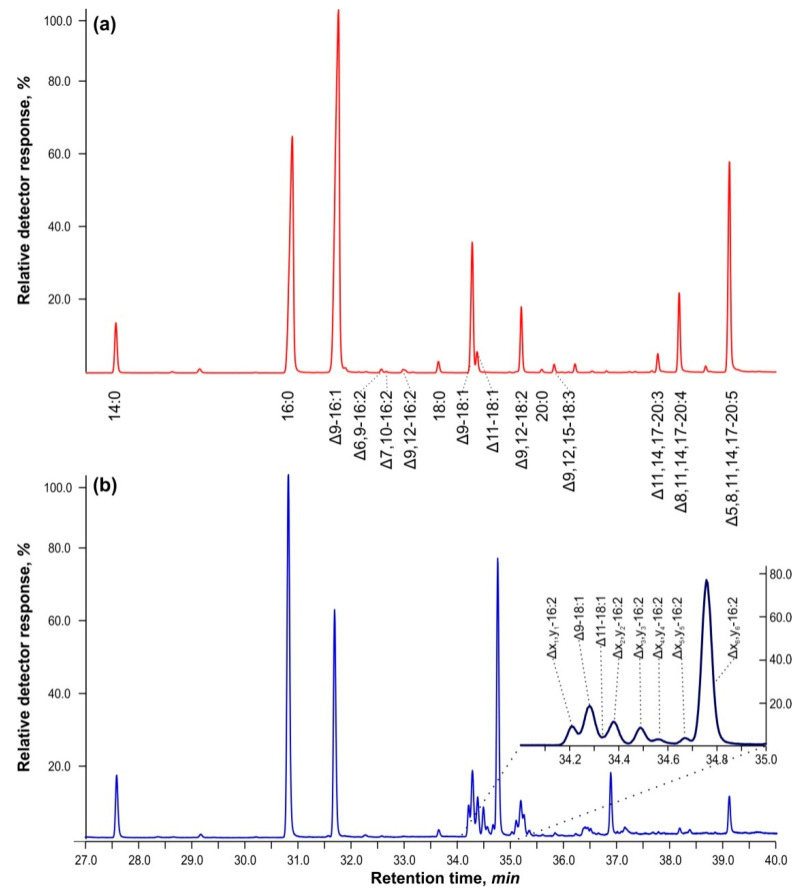
GC–MS chromatograms of the separation of fatty acid methyl esters of total lipids in cells of *Vischeria* sp. strain IPPAS C-70 (total ion current). (**a**) Control cells cultured in BBM-3N, (**b**) cells cultured with hydrogen peroxide at a final concentration of 5 mM. The insert in (**b**) details the positions of monoenoic and dienoic FAs, newly appeared in the presence of H_2_O_2_.

Figure 1 shows GC–MS chromatograms for the separation of FAMEs of total lipids in cells of *Vischeria* sp. strain IPPAS C-70 cultured in BBM-3N (Figure 1a) and cells grown in BBM-3N supplemented with 5 mM of hydrogen peroxide, H_2_O_2_ (Figure 1b). Cells exposed to H_2_O_2_ exhibited a cluster of methyl ester peaks with retention times ranging from 34 to 35 min. The mass spectra and molecular ions closely matched the library spectra of methyl esters of palmitolinoleic and Δ7,10-hexadecadienoic acids (95–98%). The mass spectra of methyl esters of unsaturated FA isomers have high homology based on double bond position, but their retention times differed by more than 3 min from those of methylene-interrupted hexadecadienes—diunsaturated FAs, in which the ethylenic bonds have the following configuration —СН_2_—CН=CН—CH_2_—CН=CН—CH_2_—. This led to the notion that these compounds may have a conjugated double bond configuration, i.e., —СН_2_—CН=CН—CH=CН—CН_2_.

It is worth noting that in BBM-3N+5 mM H_2_O_2_, the amount of unusual hexadecadienoic acids increased by 28.5% when compared to control cells cultured in BBM-3N without hydrogen peroxide.

In order to increase the biomass yield, we tried to grow *Vischeria* sp. in Tamiya medium with high N, P, Mg, and S content. But after replacement of BBM-3N with Tamiya medium, the cells were stressed and several days were required to acclimate to a new growth media and slowly resume the growth. In acclimated cells, on the 10th day of growth, regular FA desaturation was not significantly affected, but the unusual hexadecadienoic acids appeared. The concentration of unusual hexadecadienoic acids was lower than in cells exposed to H_2_O_2_; nevertheless, it reached 8% (Table 1).

### 2.2. Fatty Acids, Separated with Silver Ion TLC

To obtain a valid mass spectrum, a significant amount of the chemical in the peak is needed. Because the compounds of interest were primarily minor components, we concentrated methyl esters by separating them according to their degree of unsaturation on TLC plates coated with silica gel containing silver ions. Table 2 shows five fractions with varied retention factors and their FA compositions.

Using this procedure, a fraction with R*_f_* ≈ 0.51 was obtained, consisting of 97.8% of methylene-separated dienes with 16 and 18 carbon atoms in the chain. Although the FAs in question do not represent more than 0.1–0.5% by weight in methyl esters of total lipids (as shown in Figure 1 and Table 1), their relative proportion in this fraction increased by 10–30 times, making it possible to obtain good mass spectra of their nicotinyl ethers and DMOX derivatives.

Surprisingly, this fraction did not contain conjugated hexadecadienes. Instead, we identified them in the methyl ester fraction with R*_f_* ≈ 0.65, where monounsaturated fatty acids predominate.

The fractions with R*_f_* 0.51 and 0.65 were divided into two parts: the first was used to investigate the chromatographic properties of the detected dienes, and the second was used to determine the position and configuration of the double bonds with UV spectroscopy and mass spectrometry of their nicotinyl ethers and DMOX derivatives.

### 2.3. HPLC Diode Array Analysis Reveals a Conjugated Configuration of Double Bonds in Hexadecadienes

**Figure 2:**
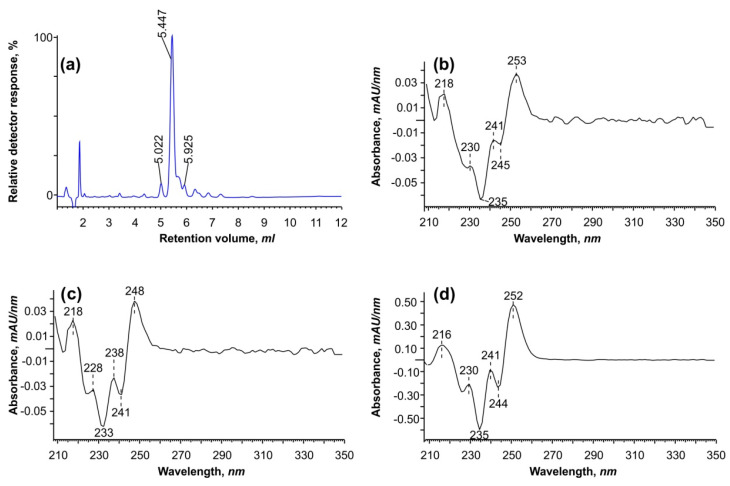
(**a**) HPLC−DAD (high-performance liquid chromatography with diode array detection) chromatogram at 234 nm of the separated free FAs from total lipids of *Vischeria* sp. strain IPPAS C-70 cells incubated with hydrogen peroxide (**a**); and the second-derivative UV spectra of individual peaks (**b**–**d**), belonging to the conjugated dienes with R_V_ 5.022, 5.447, and 5.925, respectively.

Figure 1 illustrates that the atypical dienes of interest interfere with two major peaks of oleic and *cis*-vaccenic acids. Because it is difficult to separate and purify these peaks during GC–MS analysis, we converted them back to free FAs and analyzed them using reverse-phase HPLC (Figure 2).

Using HPLC at a wavelength of 234 nm, we identified three peaks with second-derivative spectra comparable to conjugated linoleic acid isomers described in the literature [21]. The only slight difference was a “shift” at 245 and 253 nm, which is unlikely to be practically relevant because the approach did not allow us to separate all six isomers discovered by GC–MS. However, it was determined that the ethylene bonds in these dienes are conjugated. In addition, when using a fraction collector, peaks between 5 and 6.5 min were collected for additional analysis to determine the position of double bonds (see Section 2.5).

### 2.4. Chromatographic Properties of Conjugated and Methylene-Interrupted Dienes in Isothermal Analysis Modes

**Figure 3:**
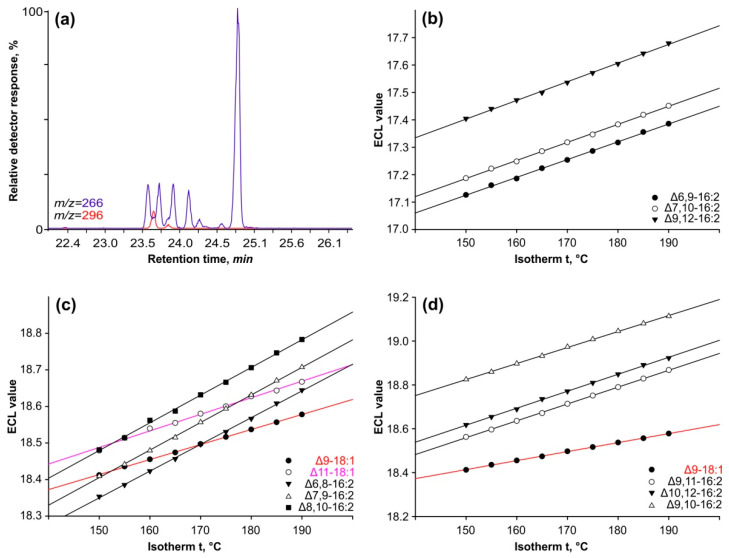
Chromatographic properties of methylene-interrupted and conjugated dienes from the fractions with R*_f_* 0.51 and 0.65 (**a**) region 22–26 min of ion chromatogram for methyl esters of conjugated hexadecadienes. Plots of equivalent chain lengths of methyl esters of octadecenoic and hexadecadienoic acids as a function of temperature, on Restec Rtx-2330 capillary column (**b**–**d**).

Retention time alone is insufficient for determining methyl esters’ chromatographic properties because it is dependent on a variety of conditions such as column temperature, gas pressure, column wear, and sample load. To address this constraint, we used a methodology that involved examining the change in equivalent chain length (ECL) across a series of isothermal analyses (Figure 3).

The ECL idea employs methyl esters of saturated FAs as reference points, with chain lengths varying by one or two carbon atoms. Their ECL equals the number of carbon atoms and has an integer value. The logarithm of these compounds’ retention time is linearly dependent on the analysis temperature; therefore, the ECL parameter more properly characterizes the methyl ester’s characteristics and has some predictive properties.

Separating *cis*-isomers of octadecenoic acids (such as oleic and *cis*-vaccenic acids) from conjugated hexadecadiene acids in isothermal studies can be challenging, as illustrated in Figure 3a. We calculated the ECL using the retention times of the peaks in the ion chromatograms indicating the molecular ions of 18:1 (*m/z* = 296) and 16:2 (M+ = 266 *m/z*).

Figure 3b shows the linear relationship between the ECLs of three methylene-interrupted hexadecadienoic acids and the temperature of analysis. Each 5-degree increase raises the ECL value by 0.0033 ± 0.0001 units for all three isomers of the double bond position. Furthermore, the difference between ECL of Δ6,9-16:2 and Δ7,10-16:2 across the entire temperature range investigated is 0.0063 ± 0.0002. As a result, altering the system of two methylene-interrupted ethylene bonds by one carbon atom causes an increase in ECL equal to the constant identified for that group of compounds. The ECL values of Δ7,10-16:2 and Δ9,12-16:2 acids varied by only 0.0235 ± 0.0005 units, closely corresponding to the predicted value. The ECL value for the Δ8,11-16:2 isomer is expected to be similar to those for the Δ7,10-16:2 and Δ9,12-16:2 isomers.

In a series of conjugated 16:2 isomers, ECL values showed a linear dependence on analysis temperature (Figure 3c). The ECLs of their peaks vary at identical intervals of 0.069 ± 0.002 units across the temperature range examined. This suggests that these isomers differ from one another by shifting the conjugated double bond system one carbon atom further from the carboxyl terminus. Furthermore, compared to methylene-separated dienes, their ECL values are 1.22 units higher.

Figure 3c depicts the corresponding chain lengths for oleic and vaccenic acids. The complete separation of these two acids from the 16:2 isomers was only possible in isotherms above 185 °C or in polythermal mode (refer to Figure 1).

The final peak’s ECL differed by 0.198 ± 0.006 from the penultimate peak, and in isometric studies, this parameter increased to values exceeding 19.11 units (Figure 3d).

In conclusion, the results presented in this section clearly suggest that there exists a group of 16:2 isomers with the conjugated system of double bonds that differ by only one carbon atom counting from the carboxyl end. Mass spectrometry of their DMOX derivatives was subsequently used to pinpoint the precise sites of these double bonds.

### 2.5. Determination of Double Bonds Positions in Methylene-Interrupted and Conjugated Hexadecadienes by Mass Spectrometry of Their Nitrogen-Containing Derivatives

**Figure 4:**
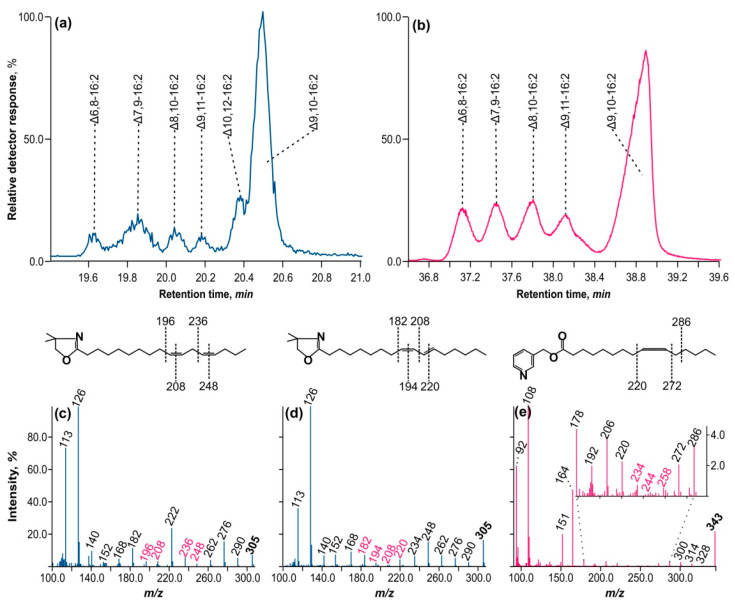
GC–MS ion chromatograms of various nitrogen-containing derivatives of hexadecadienoic acids and mass spectra of their selected peaks. (**a**) Ion chromatogram at *m/z* = 305 of DMOX derivatives of conjugated hexadecadienoic acids; (**b**) Ion chromatogram at *m/z* = 343 of nicotinyl esters of conjugated hexadecadienoic acids; (**c**) Mass spectrum of DMOX of Δ9,12-16:2 (methylene-interrupted diene); (**d**) Mass spectrum of DMOX of Δ8,10-16:2 (conjugated diene); (**e**) Mass spectrum of nicotinyl-9,10-hexadecadienoic acid (allenic configuration). Fragment ions that depict C=C binds are colored with magenta.

A fraction of conjugated hexadecadienoic acids were separated from octadecenoic acids by reverse-phase HPLC and detected at 234 nm. This fraction was subsequently converted to DMOX derivatives. Figure 4a depicts the GC–MS ion chromatogram with *m/z* = 305 separation, which reveals all six peaks that were also present in the methyl ether fraction.

Figure 4c,d show the mass spectra of DMOX derivatives of methylene-interrupted diene and hexadecadiene with a conjugated double bond. Two crucial ions with *m/z* = 113 and 126, typical of DMOX derivatives, correspond to the aliphatic chain’s second and third carbon atoms, counting from the carboxyl terminus. Starting with the ion with *m/z* = 126, a series of fragment ions can be observed that differ in mass by 14 *m/z*, corresponding to the -CH_2_- fragment of the aliphatic chain. This series is interrupted by a pair of ions with a mass difference of 12 *m/z*, suggesting that two hydrogen atoms have been lost in the aliphatic chain, resulting in —CH=CH—. Figure 4c shows two pairs: 196–208 and 236–248 *m/z*. Between fragment ions 208 and 236, there is an ion of mass 222 *m/z* that is equidistant from both, indicating the presence of a —CH_2_— group between double bonds. Following the ion *m/z* = 236, there is a chain of ions with a mass delta of 14 *m/z*. The mass spectrum is completed by ion *m/z* = 305, which differs by *15 m/z* from ion *m/z* = 290 and indicates —CH_3_ or the methyl end of the aliphatic chain. This mass spectrum matches Δ9,12-hexadecadienoic acid.

Figure 4d depicts two pairs of fragment ions, separated by 12 amu: 182–194 and 208–220. There is no 14 amu ion equidistant between 194 and 208, as in the preceding case, indicating that the ethylene bonds are conjugated. The mass spectrum corresponds to the DMOX derivative of Δ8,10-hexadecadienoic acid.

Using the specified logic for interpreting DMOX derivative mass spectra, we identified the positions of double bonds in all hexadecadienoic acids discovered throughout this study, both methylene-interrupted and conjugated. The diagnostic fragment ions are summarized in Table 3. The table displays the chromatographic properties of these acids’ methyl esters under polythermal conditions on an Rtx-2330 capillary column, as well as the features of their DMOX derivative mass spectra.

However, the peak of DMOX derivatives with a retention time of 20.55 min (refer to Figure 4a) posed difficulties because its mass spectrum had conflicting fragment ions in the region suggesting the existence of double bonds. As a result, no definitive conclusion could be drawn about their position. Based on the variations in the ECL of the methyl ester associated with this peak, it was assumed that this compound had an allene configuration of double bonds (—CH_2_—CH=C=CH—CH_2_—). Such compounds are usually identified by mass spectrometry on their nicotinyl esters.

Figure 4b shows an ion chromatogram (*m/z* = 343) of nicotinyl esters of conjugated hexadecadienoic acids. In comparison to DMOX on a highly polar capillary column, these compounds exhibit inferior chromatographic characteristics, as shown in Figure 4a. As a result, only five peaks exhibited appropriate chromatographic resolution. However, the peak of interest at 39 min was clearly distinguished from the preceding set of four peaks. Its mass spectrum is depicted in Figure 4e.

There is a prominent molecular ion at *m/z* = 343, as well as ions from the carboxyl end with a 3-pyridino moiety at *m/z* = 92, 108, 151 (the MacLafferty ion), and 164 (the third carbon atom from the carboxyl end). This is followed by a sequence of ions with a 14 amu gap up to a fragment ion at *m/z* = 234, followed by a series of ions with 10 and 14 amu gaps at 244 and 258 *m/z*, respectively, suggesting the existence of double bonds. The fragment beta ion (from the proximal end of the double bond system) with *m/z* = 220 and the alpha cleavage to the distal double bond system (in this case at *m/z* = 272) is more apparent than the series of preceding ions. There were also two ions with a 10 amu difference, 234 and 244, which appear to be the product of a structural rearrangement upon electron ionization.

These findings suggest that double bonds in the allene configuration occur at Δ9,10 carbon atoms in the aliphatic chain. The mass spectrum of the nicotinyl ester of Δ9,10-ocadecadienoic acid shows an identical fragmented pattern in the region of the double bonds [22].

## 3. Discussion

We describe FA fractions enriched in hexadecadienoic acids and show the position of double bonds in them using both mass spectrometry data from various FA derivatives and spectrophotometric data. These chemicals are conjugated and allene FAs that cells produce in response to specific adverse conditions. Some investigations have shown that conjugated FAs are formed during salt and UV stresses [2,23]. It should be noted, however, that both soil salinity and elevated UV can cause the development of reactive oxygen species (ROS) in cells, promoting oxidation and the consequent change of double bonds in acyls. At the moment, we cannot yet say definitively whether conjugated and/or allene FAs are the results of some enzymatic activity or whether they are generated under the direct action of ROS due to oxidative stress.

Apart from unique C16 FAs, H_2_O_2_ causes a substantial drop in UFAs while simultaneously increasing saturated FAs. This observation suggests that fatty acid desaturation may be inhibited either at the level of fatty acid desaturase (FAD) activity (by contact with catalytic Fe clusters) or at the level of FAD gene expression. The effect of H_2_O_2_ on FADs has not yet been thoroughly investigated, requiring additional research.

The natural C14 and C18 allenic FAs have been reported [22]. Isolation and identification approaches limit the ability to identify new and unique FAs. At the very least, gas chromatography is neither a complete nor optimal solution for screening and detecting atypical FAs [24]. Here we applied the integrated technique including TLC, HPLC, and GC–MS to search the unusual FAs. It should be noted that all conjugated and allenic FAs characterized so far belong to C18 FAs. In the present work, Δ9,10-16:2 was found in cells grown under oxidative stress (5 mM H_2_O_2_) as well as in cells grown in not-optimal Tamiya medium. Tamiya medium may cause the stress due to relatively high salt content (9 g L^−1^) and lack of sodium ions. Perhaps the atypical FA is a part of a stress-related defense mechanism.

The enzyme linoleate isomerase, which converts 9,12-18:2 to 10,12-18:2 [25], may produce Δ9,10-hexadecadienoic acid. Under stress, endoplasmic Δ12-FAD-like enzymes, which consume palmitoleic acid, may form conjugated bonds [26]. Alternative functional outcomes can be generated by Δ9-acyl-ACP desaturase, which may also possess dioxygenase activity. This enzyme oxygenates the Δ9-double bond of oleoyl (18:1Δ9)-ACP, producing a vicinal diol at the C-9 and C-10 positions with the final product erythro-9,10-dihydroxystearic acid [27].

TAGs in the seeds of several Lamiaceae species contained laballenic acid, whereas polar lipids did not. In TAGs, this acid esterified all three glycerol *sn*-positions [28]. In *Phlomis* seeds, laballenic acid was found either in TAGs or, more abundantly, in the pool of free FAs [29].

In the future, we aim to determine the biosynthetic pathway of these molecules, the class(es) of lipids in which they are located, and their potential involvement in the response to stressful environmental conditions.

## 4. Materials and Methods

### 4.1. Microalgae Strain and Growth Conditions

*Vischeria* sp. strain IPPAS C-70 (18S rRNA and ITS1-5.8S-ITS2 sequences GenBank accession numbers MN164434 and MN164431, respectively) was obtained from the Collection of microalgae and cyanobacteria IPPAS (K.A. Timiryazev Institute of Plant Physiology RAS, Moscow, Russia). The axenic culture was maintained on agarized Bold’s Basal Medium with 3-fold nitrogen (BBM-3N, Table 4) [30] in Petri dishes at 22 °C under continuous illumination with cool white luminescent lamps providing an average irradiation of 30 μmol photons m^−2^ s^−1^. For the experiments, the cells were pregrown for 10–14 days in 300 mL Erlenmeyer flasks with 100 mL of BBM-3N on an orbital shaker at room temperature under average illumination of 50 μmol photons m^−2^ s^−1^ from warm white light-emitting diodes (LEDs). Then, the culture was transferred into a glass vessel with 250 mL of BBM-3N and cultivated for 3–4 days at 29 ± 1 °C in a thermostatically controlled laboratory system for intensive cultivation [31]. Continuous illumination of 110 μmol photons m^−2^ s^−1^ was provided by warm white LEDs from both sides. Culture mixing and aeration were achieved by bubbling with sterile air enriched with 1.5–2% CO_2_. The resulting exponentially growing culture was used as an inoculum for the further experiments including stress treatments. The inoculum culture was diluted with fresh medium to optical density measured at 750 nm (OD_750_) of ~0.1–0.2, and three 225 mL aliquots were transferred to new vessels.

Oxidative stress was achieved by the addition of H_2_O_2_ (Sigma-Aldrich, St. Louis, MO, USA; H1009-100ML; 140 μL of H_2_O_2_ premixed with 25 mL of BBM-3N) to glass vessels with 225 mL of algal culture to a final concentration of 5 mM. To control samples, an equal amount (25 mL) of sterile ultrapure water was added. After 24 h of incubation, cells were collected by centrifugation and used for the analyses. 

To replace the growth medium, the inoculum of pregrown culture was centrifuged and divided in half. One half was resuspended in fresh Tamiya medium (Table 4) [32]. to optical density measured at 750 nm (OD_750_) of ~0.1–0.2, and three 250 mL aliquots were transferred to new vessels. Another part was resuspended in fresh BBM-3N and used for control. After 10 days, cells were collected and used for analyses.

### 4.2. Preparation of Free Fatty Acids

To obtain free fatty acids (FFAs) from total lipids, 500 mg of freshly precipitated microalgal cells were resuspended in 25 mL of 1M potassium hydroxide solution in 80% aqueous ethanol. The suspension was transferred to a 50 mL round-bottomed flask, sealed with a reflux condenser, and incubated in a sand bath at 80 °C for 1 h. After this time, the flask was left to cool to room temperature, the contents were transferred into a 50 mL Eppendorf^®^ tube, and three 15 mL portions of *n*-hexane were used to extract unsaponifiable components such as carotenoids, sterols, etc. The emulsion was vortexed for 60 s, and then the upper hexane phase was separated by centrifugation at 2300× *g* for 5 min. The upper phase was removed, 20% aqueous sulfuric acid was added to the residue in a test tube to change the pH to a slightly acidic reaction, monitored by indicator paper, and FFAs were extracted three times with 15 mL of *n*-hexane. The resulting fractions were combined and then evaporated to dryness in vacuum, and the dry residue was redissolved in 5 mL of *n*-hexane and stored at −20 °C for further analysis.

### 4.3. Preparation of Fatty Acids Methyl Esters from FFAs

In order to prepare FAMEs, 5 mL of a solution of free FAs with a concentration of 10 mg mL^−1^ in *n*-hexane was transferred to a 50 mL glass round-bottom flask and the solvent was removed in vacuum. Then 10 mL of a 10% solution of acetyl chloride in absolute methanol was added to the dry residue. The flask was closed with a reflux condenser connected to a circulation cooler and kept in a sand bath for 1 h at a boiling point of the solution of ca. 75 °C. After the flask was left to cool to room temperature, the solution was transferred to a 50 mL test tube, 5 mL of water was added, and FAMEs were extracted three times with *n*-hexane. The solvent was evaporated on a rotary vacuum evaporator, and FAMEs were redissolved in 5 mL of heptane and stored at −20 °C until further work or analysis.

### 4.4. Concentration of Minor Fatty Acids by Silver TLC

The methyl esters of fatty acids from the total lipids were separated based on their degree of unsaturation using Fluka TLC plates measuring 10 × 10 cm. The plates were preincubated in a 3% methanol solution of silver nitrate for 15 min and activated in an oven at 120 °C for an hour. The mobile phase used was chloroform: acetone 100:6 *v*/*v*. Zones containing fatty acid methyl esters (FAMEs) with retention factors (Rf) of 0.03, 0.07, 0.15, 0.51, 0.65, and 0.83 were visualized by spraying the plate with a 0.001% solution of 2,7-dichlorofluorescein in 50% aqueous methanol under UV light at 365 nm. The sorbent containing FAMEs from each zone was carefully transferred into individual 2 mL tubes. Then, 500 μL of water and 1 mL of a 1:1 *v*/*v n*-hexane: diethyl ether mixture were added. The tubes were vortexed for 30 s, and the upper phase was separated by centrifugation for 3 min at 2300× *g*. Afterward, the upper layer was collected, evaporated to dryness in a stream of argon, and the dry residue was redissolved in heptane.

### 4.5. Preparation of Acid Chlorides of Fatty Acids from Free FAs

The synthesis of nitrogen-containing derivatives of fatty acids was carried out through their acid chlorides, which were obtained by heating 10 mg of free fatty acids (see Section 4.2) with 500 μL of oxalyl chloride in a 2 mL glass vial. The mixture was then kept at 55 °C for 1 h. Afterward, the residue of oxalic acid chloride was evaporated to dryness in a stream of argon.

### 4.6. Preparation of Dimethyloxyzoline Fatty Acid Derivatives (DMOX)

To 5 mg of fatty acid chlorides (see Section 4.5), 750 μL of a solution of 2-amino-2-methyl-1-propyl alcohol in dry dichloromethane (15 mg mL^−1^) was added and left for an hour at 50 °C, after which the solvent was evaporated to dryness in flow of argon and 500 μL of trifluoroacetic anhydride was added to the dry residue in the vial and, again, it was kept in a solid thermostat at 50 °C for 1 h. After the time had passed, the anhydride was evaporated to dryness in a stream of argon. Next, 300 μL of water and 1 mL of *n*-hexane were added to the dry residue in the vial. The mixture was vortexed for 30 s, and after phase separation, the upper layer was collected in a clean vial. Then, 50 mg of anhydrous sodium sulfate was added to the collected solution of the resulting derivatives. The solution was stored at +4 °C until further analysis.

### 4.7. Preparation of 3-Pyridylcarbinol (Nicotinyl) Esters of Fatty Acids

A total of 5 mg of fatty acid chlorides (see Section 4.5) was redissolved in a volume of 300 μL of a 20% solution of 3-hydroxymethylpyridine in dry acetonitrile in a 2 mL glass vial with a screw cap, then kept for 1 min at 100 °C in a heating block. Acetonitrile was evaporated to dryness in a stream of argon at 55 °C, after which nicotinyl ethers were extracted with 1 mL of a mixture of *n*-hexane: diethyl ether 1:1 *v*/*v*. To purify the ethers, their solution was applied with a microsyringe to a cartridge with Junior Si silica gel. The cartridge was then washed with 5 mL of a mixture of *n*-hexane: diethyl ether 1:1 *v*/*v*. The solvents were evaporated to dryness in a stream of argon, and the dry residue was redissolved in 500 μL of heptane.

### 4.8. HPLC Diode Array Analyses of Free Conjugated Hexadecadienoic Acids

Conjugated hexadecadienoic acids in total lipid fatty acids were detected using reverse-phase HPLC, following the method of [10] with slight modifications. A total of 5 mg of free fatty acids (see Section 4.2) was redissolved in 1 mL of 0.14% glacial acetic acid in acetonitrile, *v*/*v*. Then, 20 μL of this solution was injected into the HPLC system. The free fatty acids were separated using a Shimadzu Prominence LC-20AP liquid chromatograph equipped with an SPD M-20A diode matrix detector on an MN Nucleodur HTec C18 column (250 mm × 4.6 mm, 5 μm) in isocratic mode. The mobile phase consisted of acetonitrile, water, and glacial acetic acid in a ratio of 70:30:0.12 *v*/*v*/*v*, and was run at a flow rate of 1 mL min^−1^. The chromatogram was recorded at 234 nm with a data collection frequency of 0.5 Hz. LabSolutions ver. 5.73 software (Shimadzu Co., Tokyo, Japan) was used to generate second-derivative UV spectra.

### 4.9. GC–MS Analysis of Fatty Acids Derivatives

The capillary GC method was used to separate fatty acid methyl esters of total lipids, as well as nicotinyl and DMOX derivatives. The separation was performed on an Agilent 7890A chromatograph with an Agilent 5975C quadrupole mass spectrometric detector (Agilent Technology Inc., Santa Clara, CA, USA). The chromatograph was equipped with a 60 m HP-88 capillary column with an internal diameter of 0.25 mm and a stationary phase thickness (88%(Cyanopropyl)aryl-polysiloxane) 0.2 µm. The following conditions were used.

For FAMEs, the temperature profile started at 60 °C for 8 min, followed by a ramp rate of 7 °C per min until reaching 175 °C for 5 min, and finally ramping up to 245 °C at a rate of 9 °C per min for 60 min. Finally, the temperature was increased by 9 °C per min until it reached 245 °C for 60 min. The injector temperature was set to 260 °C and the sample was introduced with a split ratio of 20:1, the ion source temperature was set to 230 °C, the quadrupole was set to 150, and the ionization energy was set to 70 eV. Chromatograms were recorded using the total ion current. The peaks of methyl esters were identified by matching their chromatographic parameters (retention time, relative retention time) and mass spectra with the NIST database using Agilent MSD ChemStation v. 4.0.3 software (Agilent Technology Systems, Santa Clara, CA, USA).

For DMOX, the initial temperature was set at 130 °C for 1 min. It was then increased at a rate of 6.5 °C per min until it reached 170 °C. The temperature was then increased at a rate of 2.75 °C per min until it reached 215 °C for 25 min. Finally, the temperature was increased at a rate of 5 °C per min until it reached 240 °C for 60 min. The carrier gas used was helium at a flow rate of 1 mL min^−1^. All other GC–MS parameters were identical to the FAME preparation method.

The separation of nicotinyl esters of FAs was carried out using the following conditions: a helium carrier gas flow of 1.5 mL min^−1^, an initial oven temperature of 130 °C for 1 min, followed by a gradual increase to 185 °C at a rate of 15 °C per min, an 8 min plateau, and a further increase to 245 °C at a rate of 5 °C per min with a hold at 245 °C for 45 min. The total analysis time was 70 min.

The mass range scanned in all methods was 50–550 *m/z*. After subtracting the zero-line noise using the ChemStation program, the mass spectra of DMOX derivatives and nicotinyl esters were interpreted with AMDIS and OpenChrom v. 1.5.0 software (https://www.openchrom.net; Accessed on 12 March 2024) [33].

### 4.10. GC–MS Determination of Equivalent Chain Length (ECL) of Fatty Acids

To the FAMEs solution from fractions with retention factors of 0.65 and 0.51 (refer to Section 4.2), margaric acid methyl ester was added. Additionally, a solution of a mixture of Supelco GLC-100 methyl esters was also added as a source of methyl stearate and methyl nonadecanoate, which are necessary for the most accurate calculation of the ECL of UFAs eluted between FAMEs with an integer ECL value.

The ECL of methyl esters of methylene-interrupted and conjugated hexadecadienoic FAs was determined using a Crystal 5000 NP gas chromatograph. An AS-2M single-port autosampler, a Chromatec Crystal MSD mass spectrometric quadrupole detector, and a 60 m capillary column Restek Rtx-2330 with an internal diameter of 0.25 mm and a stationary phase layer thickness of 0.2 µm were used for the analysis. The injector temperature was set at 260 °C, and the sample was injected at a split ratio of 5:1. The analyses were conducted isothermally at temperatures ranging from 150 to 190 °C in 5-degree increments. The carrier gas used was helium at a flow rate of 1 mL min^−1^. The ion source temperature of MSD was set to 230 °C, the quadrupole was set to 150 °C, and the ionization energy was set to 70 eV.

Chromatograms were recorded in total ion current mode, scanning masses in the range of 50–550 *m/z*. To determine the exact retention times of the compounds studied, ion chromatograms were extracted from molecular ions with *m/z* = 284 for 17:0, *m/z* = 266 for hexadecadienoic FAs, *m/z* = 298 for methyl stearate, *m/z* = 296 for octadecenoic FAs acids, and *m/z* = 312 for nonadecanoic acid methyl ester using AMDIS software v. 2.66. (http://www.amdis.net; accessed on 12 March 2024)

The formula used to calculate the equivalent carbon chain length is as follows:

ECL(x) = z + ((log t’R(x) − log t’R(z))/(log t’R(z+1) − log t’R(z))),

where t’R is retention time of the analyte; z is the carbon number in the saturated FAME eluting immediately before the analyte of interest; x, and z + 1 is the number of carbons in the saturated FAME eluting immediately after analyte x.

## 5. Conclusions

This work presents an integrated approach for the isolation, purification, concentration, derivatization, and analysis of hexadecadienoic fatty acids, including methylene-interrupted, conjugated, and allenic FAs. We described the chromatographic, UV spectroscopy properties of their methyl and nicotinyl esters, DMOX derivatives, and determined the position of the double bonds. The microalga *Vischeria* sp. strain IPPAS C-70 can accumulate up to nine isomers of hexadecadienoic acids under different cultivation conditions. A significant proportion of these isomers is 9,10-allene hexadecadienoate, together with a series of the position of the conjugated system of ethylene bonds, differing by a “shift” of one carbon atom for each other. We hypothesize that appearance of dienes and allenes among FAs may be the consequence of oxidative stress due to H_2_O_2_. In the future, we intend to identify these compounds’ biosynthesis process, the class(es) of lipids in which they are located, and their possible role in the response to stressful environmental situations. It will also be intriguing to investigate other stressful situations having an oxidative effect, such as salt, high light, and temperature stress, as well as the effects of toxic metals. Future research should look for similar FAs in other organisms subjected to environmental stresses.

While the pharmaceutical and nutritional importance of conjugated C18 linoleic acid (CLA) has been intensively studied, and its potent beneficial effects, including antitumor, antiobese, antiatherogenic, and antidiabetic activities, have been reported [34], nothing is known about the impact of unusual C16-conjugated FAs. These isomers are found in minor quantities in cellular lipids, yet their functional role in cellular physiology and possible biotechnological potential has yet to be investigated.

## Figures and Tables

**Table 1:** The content of FAMES in individual areas (mass %) in cells of *Vischeria* sp. strain IPPAS C-70 grown in BBM-3N, BBM-3N supplemented with 5 mM hydrogen peroxide for 24 h (+H_2_O_2_), and in Tamiya medium (T) for 10 days.

FAs	BBM	T	+H_2_O_2_
14:0	3.2	5.8	4.9
16:0	23.2	16.4	31.2
Δ9-16:1	38.6	36.1	17.6
Δ11-16:1	0.4	0.4	tr.
Δ7,10-16:2	0.2	0.7	tr.
Δ9,12-16:2	0.3	2.1	tr.
18:0	0.7	0.4	0.5
Δ9-18:1	7.9	2.5	5.3
Δ11-18:1	1.3	tr.	tr.
UHDs	-	8.1	28.5
Δ9,12-18:2	3.8	5.5	2.9
Δ6,9,12-18:3	0.5	tr.	tr.
Δ9,12,15-18:3	0.5	0.8	tr.
UODs	-	2.3	5.9
Δ8,11,14-20:3	1.1	tr.	tr.
Δ5,8,11,14-20:4	4.6	2.4	0.4
Δ5,8,11,14,17-20:5	13.7	16.5	2.9

UHDs—sum of unusual hexadecadienoic acids; UODs—sum of unusual octadecadienoic acids; tr.—FAs identify in trace amount (<0.05%); -—FAs were not identified in a sample. Individual peaks were identified with Agilent MSD Chem-Station 4.0.3 software and the NIST library.

**Table 2:** Fatty acid composition of methyl ester fractions of total lipids of *Vischeria* sp. strain IPPAS C-70 exposed to 5 mM of hydrogen peroxide for 24 h, obtained as a result of their separation on a TLC plate with silver nitrate.

Fatty Acid, *mas.%*	Fraction R*_f_* Value					
	0.03	0.07	0.15	0.51	0.65	0.83
10:0						1.0
12:0						1.1
14:0						26.0
16:0						70.3
18:0						0.9
Δ6-16:1					0.3	
Δ9-16:1				tr.	90.2	tr.
Δ11-16:1					0.3	
Δ9-18:1				2.2	3.0	tr.
Δ11-18:1					0.5	
Δ6,9-16:2				8.9		
Δ7,10-16:2				1.3		
Δ9,12-16:2				42.0		
Δ6,8-16:2					0.6	
Δ7,9-16:2					0.4	
Δ8,10-16:2					0.7	
Δ9,11-16:2					0.8	
Δ10,12-16:2					0.6	
Δ9,10-16:2					1.5	
Δ8,11-18:2				2.0		
Δ9,12-18:2				43.6		
UODs *					1.1	
Δ7,10,13-16:3			12.6			
Δ6,9,12-18:3			7.9			
Δ9,12,15-18:3			79.5			
Δ6,9,12,15-18:4		14.8				
Δ8,11,14,17-20:4	1.1	79.9				
Δ5,8,11,14,17-20:5	98.9	5.3				
Others **						1.0

* UODs comprised four isomers of conjugated double bonds position of octadecadienoic acids. ** Others also contained minor FAs, 11:0, 13:0, 15:0, *iso*-14:0, and *anteiso*-14:0, in quantities of no more than 0.3%.

**Table 3:** Chromatographic properties of methyl esters of methylene-interrupted and conjugated hexadecadienes, as well as diagnostic fragment ions of their DMOX derivatives and nicotinyl ethers, indicating the positions of double bonds.

Fatty Acid	FAMEs Chromatographic Properties *			DMOX Derivatives Fragment Ions	Nicotinyl Esters, Fragment Ions	
				M_+_ = 305 *m/z*	M_+_ = 343 *m/z*	
	R^T^ *min*	RR_T18:0_ **	ECL Value	Δ12 *a.e.m.*	Δ40 *a.e.m.*	Δ26 *a.e.m.*
Methylene-interrupted hexadecadienes						
Δ6,9-16:2	20.217	0.880	17.17	180–167	232–192	232–218
				206–194	258–218	258–232
Δ7,10-16:2	20.380	0.906	17.23	180–168	246–206	232–206
				220–208	272–232	272–246
Δ9,12-16:2	20.989	0.933	17.46	208–196	260–220	260–234
				248–234	300–260	300–274
Conjugated hexadecadienes ***						
Δ6,8-16:2	23.556	1.053	18.46	166–154	244–192	
				192–180		
Δ7,9-16:2	23.719	1.060	18.52	180–168	258–206	
				206–194		
Δ8,10-16:2	23.916	1.069	18.59	194–182	272–220	
				220–208		
Δ9,11-16:2	24.139	1.079	18.67	208–196	286–234	
				234–222		
Δ10,12-16:2	24.294	1.086	18.73	222–210	Not separated	
				248–236		
Allenic hexadecadiene						
Δ9,10-16:2	24.603	1.099	18.84	-	286-220 ****	

* Based on the data from the polytherm mode of analysis (for conditions, see Section 4.10). ** Methyl stearate had retention time of 22.491 min. *** Only the pair of ions with a gap of 52 *m/z* at either end of the conjugated double bond system is shown in the table. **** Diagnostic gap for allenic double bonds (see details in the text).

**Table 4:** The composition of BBM-3N and Tamiya media.

Components, g L^−1^	BBM-3N	Tamiya
NaNO_3_	0.75	-
KNO_3_	-	5
KH_2_PO_4_	0.175	1.25
K_2_HPO_4_ (×3H_2_O)	0.075	-
MgSO_4_ × 7H_2_O	0.075	2.5
CaCl_2_ × 2H_2_O	0.025	-
NaCl	0.025	-
Fe + EDTA solution: 30.2 g EDTA-Na_2_ was dissolved in 134 mL of 1M KOH. This solution was diluted with 500 mL of distilled H_2_O, and 24.9 g of FeSO_4_ × 7H_2_O was added, and the volume was adjusted to 1 l with distilled H_2_O (the latter solution requires overnight bubbling with air through NaOH solution to avoid reaction with CO_2_	1 mL	1 mL
Microelements (g L^−1^): H_3_BO_3_ − 2.86; MnCl_2_ × 4H_2_O − 1.81; ZnSO_4_ × 7H_2_O − 0.222; MoO_3_ − 0.018; NH_4_VO_3_ − 0.023	1 mL	1 mL

## Data Availability

Data are contained within the article.

## Abbreviations

BBM-3N	Bold’s Basal Medium with 3-fold nitrogen
CLA	Conjugated linoleic acid
DMOX	4,4-Dimethyloxazoline derivatives
ECL	Equivalent chain length
EPA	Eicosapentaenoic acid
FA	Fatty acids
FAD	Fatty acid desaturase
FAMEs	Fatty acids methyl esters
FFAs	Free fatty acids
GC–MS	Gas chromatography–mass spectrometry
HPLC	High-performance liquid chromatography
HPLC-DAD	High-performance liquid chromatography with diode array detection
LC-PUFAs	Long chain polyunsaturated fatty acids
LEDs	Light-emitting diodes
OD_750_	Optical density measured at 750 nm
R*_f_*	Retention factor
ROS	Reactive oxygen species
RRt	Relative retention time
Rt	Retention time
TAGs	Triacylglycerols
TLC	Thin-layer chromatography
UFAs	Unsaturated fatty acids
UHDs	Sum of unusual hexadecadienoic acids
UODs	Sum of unusual octadecadienoic acids
